# The Unusual Journey of a Pericardial Drainage Catheter in Pentalogy of Cantrell

**DOI:** 10.1155/2021/2109934

**Published:** 2021-07-08

**Authors:** Margaret P. Ivy, Gareth J. Morgan, Jenny E. Zablah

**Affiliations:** Department of Pediatric Cardiology, Children's Hospital Colorado, Aurora, CO, USA

## Abstract

A 4-month-old male infant diagnosed with Pentalogy of Cantrell presented to the cardiac catheterization laboratory with a large pericardial effusion. During an urgent pericardial drain placement, the patient, whose prior hemodynamics and clinical findings had suggested a noncritical cardiac lesion, had a profound desaturation, with echocardiography suggesting minimal or no flow across the right ventricular outflow tract (RVOT). The position of the drainage catheter on fluoroscopy and echocardiography suggested that the spell was being caused by obstruction of the main pulmonary artery (MPA) by the pericardial drain. After partially withdrawing the drain to reposition it, there was immediate resolution of the hypoxemia, and echocardiography once again showed adequate flow across the outflow tract.

## 1. Introduction

Pentalogy of Cantrell (PC) is a rare congenital condition with an estimated incidence of 5.5 in 1 million live births [1]. The exact cause of PC is unknown, but as described by Cantrell et al. in 1958, complete PC is characterized by five anomalies: midline supraumbilical abdominal wall defect (e.g., omphalocele or gastroschisis), a deficiency of the anterior diaphragm (anterior diaphragmatic hernia or hypoplastic diaphragm), a defect of the lower sternum or absent sternum, a defect of the diaphragmatic pericardium (ectopia cordis), and congenital intracardiac defects (such as Tetralogy of Fallot) [2]. Most reported cases of PC are incomplete; not having all of the characteristics. PC has a very high mortality rate with prognosis depending on the severity of the congenital defects. The most distinctive defects in patients with PC are omphalocele, which has an independent estimated incidence of approximately 1 in 4000 births, and ectopia cordis, whose estimated incidence is 1 to 65 in 200,000 births [3, 4]. The pericardium is absent in approximately 75% of PC with associated intracardiac defects [5]. The pericardial defect makes the development of a hemodynamically significant pericardial effusion unusual, as its communication with the parietal membrane surrounding the abdominal organs allows complete decompression and redistribution of fluid to the omphalocele sac.

Approximately 80% of patients with PC also have congenital heart disease, such as Tetralogy of Fallot, which was the case in our patient [6]. Tetralogy of Fallot (TOF) occurs in 3 of every 10,000 live births and is the commonest congenital cardiac lesion causing cyanosis [7]. TOF has four characteristic morphological findings: a ventricular septal defect (VSD), stenosis of the pulmonary valve and right ventricular outflow tract, an aorta overriding the VSD, and right ventricle hypertrophy. These features are in essence the result of anterocephalad deviation of the outlet portion of the ventricular septum, which minimizes the flow from the heart into the RVOT during development and causes underdevelopment of the outflow tract, pulmonary valve, and pulmonary arteries. Patients with TOF often experience hypercyanotic spells, commonly referred to as “tet spells.” These are characterized by a significant drop in oxygen saturations as a result of acutely decreased flow out of the RVOT probably due to the vasoconstriction of the pulmonary arterial bed and limitation of flow through the outflow tract due to thickened muscle below the pulmonary valve [7]. “Spells” are triggered by many factors that decrease the right heart preload and increase the afterload, including pericardial effusions.

## 2. Case Presentation

A 4-month-old male was born emergently by caesarian section at 32 weeks gestation. He was immediately intubated at birth due to hypoxemia. At 28 weeks gestation, a fetal ultrasound had revealed a large ventral wall defect and fetal echocardiography revealed tetralogy of Fallot. Fetal MRI revealed deficiency of the central tendon/anterior diaphragm with a small portion of the liver and small bowel loops protruding into the pericardial space, confirming findings compatible with pentalogy of Cantrell. Postnatal echocardiography confirmed the cardiac diagnosis of tetralogy of Fallot. There was not significant RVOT obstruction with minimal dynamic obstruction, normal-size pulmonary valve, and pulmonary arteries. His saturations were below 80 on 50% FiO_2_, due mostly to ventilatory and respiratory issues related to the diaphragm and ventral wall defect.

**Figure 1:**
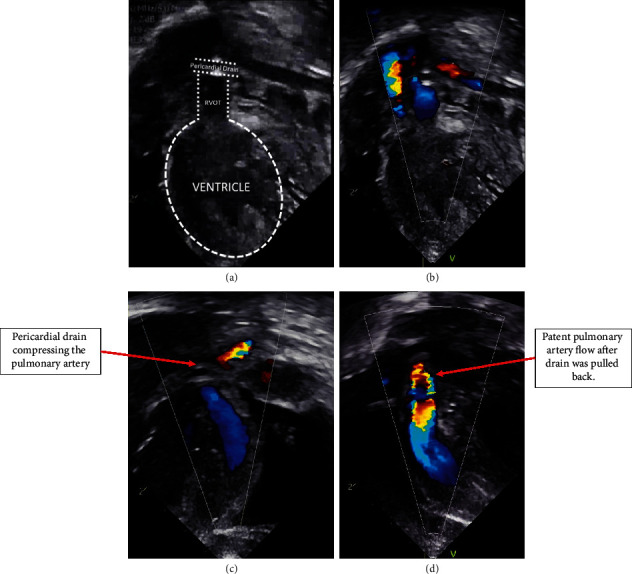
Echocardiogram on the day of the procedure demonstrating a large pericardial effusion. (a) Diagram of the echocardiographic view of the right ventricle and the pulmonary artery used to determine the compression caused by the drain. (b, c) Color Doppler echocardiogram, with and without labels, of the flow obstruction in the pulmonary artery, evident by no blood flow (color) seen distal to the drain. (d) There is improvement of the pulmonary artery flow after the drain was retracted, showing blood flow (color) along all the pulmonary artery, when compared with prior panels.

**Figure 2:**
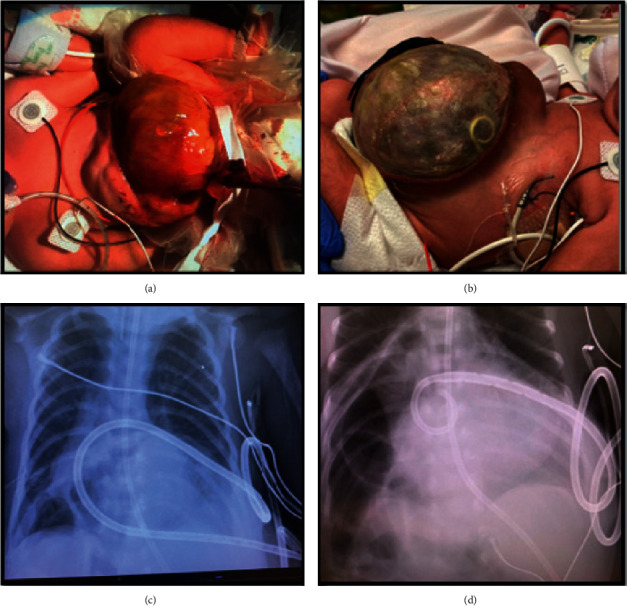
Omphalocele and drain images: (a) the patient's omphalocele at birth. (b) The Pigtail catheter placed in the pericardium is seen in the omphalocele sac, demonstrating continuity of the pericardium with the abdominal cavity. (c) Pericardial drain is seen in chest X-ray coursing around the cardiac silhouette and into the omphalocele. (d) The pigtail is retracted 2 cm with adequate draining and outside the omphalocele.

An echocardiogram at 20 days after birth was notable for a large, circumferential pericardial effusion without echocardiographic or clinical evidence of tamponade, but his saturations were worsening, requiring increased ventilatory support in the setting of this new effusion. As a result, the patient was referred for pericardiocentesis due to concern for potential hemodynamic compromise. The patient weighed 2.8 kg and was still intubated. A total of 100 mL serous fluid was acutely drained from the pericardial space, and an 8 F Pigtail drain was inserted with the standard technique from an apical approach, with position in the pericardium confirmed by echocardiography and fluoroscopy. Despite this, the patient immediately demonstrated significant refractory hypoxemia with oxygen saturations dipping into 40s. The anesthesia team replaced the endotracheal tube and administered phenylephrine boluses given concerns for a profound hypercyanotic spell. Emergent echocardiography suggested poor antegrade flow across the pulmonary valve. On continued review of the echocardiographic images, we became concerned that the main pulmonary artery was being compressed by the pericardial drain. The drain appeared to cross within the pericardial space anterior to the RVOT outflow causing obstructive compression. Once the drain was partially retracted, the hypoxia resolved immediately. Echocardiography consequently demonstrated unobstructed antegrade flow through the pulmonary artery (Figure 1). Next morning, the drain was seen in the omphalocele which was consistent with his chest X-ray. The drain was pulled further back 2 cm and sutured in place without any other complications (Figure 2). The drain was removed 2 days later without recurrence of the pericardial effusion.

## 3. Discussion

In normal subjects without omphalocele and/or diaphragmatic defects, separate membranous sacs contain the heart (pericardium), lungs (pleura), and the abdominal viscera (peritoneum) which allows for physiologic changes in intrathoracic pressures with respiration. Disorders of septation of the abdomen and thorax such as Pentalogy of Cantrell may result in continuity between the pericardial and peritoneal cavities [8].

Diaphragmatic defects allow abdominal organs, such as the liver, to herniate up into the chest near the heart. As a result, the pressure gradients that allow for venous return to the right ventricle are nonexistent during inhalation, encouraging decreased preload in the right ventricle, which may worsen hypercyanotic spells in children with TOF when found in combination.

In patients with abdominal wall defects, usually the abnormal abdominal cavity continuity allows excess pericardial fluid to disperse throughout the chest and into the abdominal cavity, so these patients will rarely have a hemodynamically significant pericardial effusion. It also, however, allows the reverse to occur with ascitic fluid from the peritoneum dispersing into the pericardial space. In this case, the patient had a large pericardial effusion retaining a significant localized collection fluid around the heart, despite not having a defined pericardium.

When the patient developed hypoxemia during the pericardial drain placement, it was initially assumed that the patient was experiencing a hypercyanotic spell, after ruling out mechanical issues with ventilation. The differential diagnoses considered for profound hypoxemia included poor ventilation from a dislodged endotracheal tube (so it was replaced), acidosis, a ventilator issue, or a typical hypercyanotic spell after a significant hemodynamic change with the pericardiocentesis. It was not initially assumed that the drain was compressing the pulmonary artery. Although the MPA is within the bounds of the pericardium, the usual limits of the pericardial space are just proximal to the pulmonary artery bifurcation. It is, therefore, very rare, in usual anatomy, for a drainage catheter to take a course that brings it over the great vessels anteriorly. Despite many potential complications associated with pericardiocentesis, compression of the pulmonary artery has not been reported [9].

This unusual anatomic relationship was further illustrated when on visual inspection of the patients' omphalocele, the pigtail drain had eventually passed into this peritoneal portion of the sac and lay in full view on the left lateral surface of the abdominal viscera.

With normal pericardial anatomy, this is a very unlikely but not impossible complication which may occur in patients with percutaneously placed pericardial drains, but more likely with surgically placed mediastinal drains in the postoperative period. Unusual complications secondary to common procedures are possible, and we need to be aware to intervene timely.

## Data Availability

No data were used to support this study.
